# The American Medical Association

**Published:** 1888-03

**Authors:** 


### THE AMERICAN MEDICAL ASSOCIATION.

The annual meeting of the American Medical Association this year will be held in Cincinnati, on the first Tuesday in May. Special efforts are being made to have it a large and representative meeting of the medical profession of the whole country. The central location of the place of meeting, and the season of the year, as well as the well-known hospitality of the people of Cincinnati, will unite to make the occasion interesting and profitable professionally and enjoyable socially.

If the national societies representing the various specialties be properly represented at the meeting of the American Medical Association, as they should be, and participate in the work of the general sessions, as well as in its different sections, it will prove alike advantageous to the Association and to the special societies by bringing into more direct contact the general practitioner and the specialist, who seem yearly to be drifting further apart. Their interests are mutual and supplemental, and the growing tendency of recent years towards isolation is detrimental to the best interests of both. Whatever cause or causes may have led to such tendency the result is unfortunate.

The general practitioner needs and desires direct personal contact with the specialist whose special course of study and restricted sphere of work have made him deft and an expert adviser in his particular field ; the specialist, whose life is devoted to work in that circumscribed field, must recognize the fact that the limitations to which he confines his work, whilst bringing expertness, have a narrowing tendency mentally, as well. Contact with those engaged in the general work of the profession serves to diminish that tendency, and thus such reunions are mutually advantageous. It is not sufficient to gainsay this to allege that the specialist should be well qualified as a general practitioner, besides being an expert specialist. All will admit its truth, but desirable as it is that such should be the case, it is patent to the observing medical man that the tendency of the last few years has been markedly in a different direction, and unless some counterbalancing influence be exerted which shall neutralize that tendency, the result in the near future will likely be mutually unsatisfactory. Since the American Medical Association must be recognized as the organization that is most truly representative of the whole medical profession of our country, and its annual meetings, with general sessions for work which interests the entire profession, and sectional meetings designed for the special work of the different departments of the profession, furnish ample opportunity for all to meet, it seems most desirable that these opportunities should not be as much neglected as they have been for the last few years. Shall not the next meeting of the Association witness a radical change for the better in that respect?

